# Serum triglyceride levels are associated with recurrence in patients with acute hypertriglyceridemic pancreatitis

**DOI:** 10.3389/fmed.2023.1079637

**Published:** 2023-03-15

**Authors:** Langyi Guan, Ling Ding, Jianhua Wan, Liang Xia, Wenhua He, Huifang Xiong, Lingyu Luo, Nonghua Lu, Yin Zhu

**Affiliations:** Department of Gastroenterology, Digestive Disease Hospital, The First Affiliated Hospital of Nanchang University, Nanchang, Jiangxi Province, China

**Keywords:** acute pancreatitis, recurrence, risk factor, hypertriglyceridemia, follow-up

## Abstract

**Aim:**

To analyze the clinical profile of patients with acute hypertriglyceridemic pancreatitis (HTGP) and explore risk factors for recurrence.

**Methods:**

A retrospective observational study was conducted in patients who experienced an attack of HTGP for the first time. Patients were followed until the recurrence of acute pancreatitis (AP) or 1 year. The detailed clinical profile was compared between patients with or without recurrence. Multivariate logistic regression analysis was conducted to explore independent risk factors for recurrence.

**Results:**

A total of 108 HTGP patients were included in this study with 73.1% being male, and the median age being 37 (interquartile range, IQR, 30.3–44.8) years. Recurrence occurred in 70 patients (64.8%). Compared with the nonrecurrent group, serum triglyceride (TG) levels before discharge [4.1 (2.8,6.3) mmol/L vs. 2.9 (2.2,4.2) mmol/L; *p* = 0.002], at 1 month [3.7 (2.3,9.7) mmol/L vs. 2.0 (1.4,2.7) mmol/L; *p* = 0.001], at 6 months [6.1 (3.1,13.1) mmol/L vs. 2.5 (1.1,3.5) mmol/L; *p* = 0.003] and 12 months [9.6 (3.5,20.0) mmol/L vs. 2.7 (1.6,5.5) mmol/L; *p* = 0.001] after discharge were higher in the recurrent group. Poor control of TG levels (TG > 3.1 mmol/l) at the 1-month follow-up after discharge and a high Charlson’s Comorbidity Index score (≥ 2 points) increased the risk of recurrence of HTGP.

**Conclusion:**

High TG levels during follow-up and Charlson’s Comorbidity Index score were independently associated with recurrence in patients with HTGP.

## Introduction

Acute pancreatitis (AP), an inflammatory condition of the pancreas, has become one of the most common acute gastrointestinal disorders in recent years. The overall incidence of AP has increased by 3.07% per year (1, 2), leading to an increasing burden on healthcare services. Overall AP-related mortality is approximately 5% (3–5). Severe acute pancreatitis (SAP) attacks develop in 20% of patients, with a substantial mortality risk up to 20–40% (6, 7). The etiology of AP is diverse, including biliary disease, hypertriglyceridemia (HTG), alcohol consumption, hyperparathyroidism, steroid use and other rare etiologies (8, 9). There are obvious differences in the etiology of AP due to differences in races, regions, and economic and health conditions (10). The incidence of hypertriglyceridemic pancreatitis (HTGP) has been increasing for a number of years (11), accounting for 0.36–25.6% of all AP cases (11–13). In some regions of China, HTG is currently the second most common cause of AP (12).

Generally, AP is a self-limiting process, and a small number of patients will have repeated attacks, known as recurrent acute pancreatitis (RAP). The pooled incidence of RAP can reach 10–30% (14–16). RAP can further lead to chronic pancreatitis (CP), causing endocrine and exocrine dysfunction of the pancreas and affecting peoples work and even normal life activities (2). It is crucial to accurately identify the cause of RAP and etiological treatment. Recent studies have shown that the recurrence rate of HTGP is high, reaching 30.1–32% (17, 18). This phenomenon may be related to the persistence of certain factors (such as drinking, diabetes, obesity and other factors) that cause to secondary dyslipidemia, poor TG control or genetic susceptibility (19–21). Prior studies mostly focused on the risk factors for recurrence of alcoholic and biliary pancreatitis (22). Studies have revealed that reducing alcohol consumption and cholecystectomy can be a way to diminish the risk of recurrent AP. Other studies mainly assessed the severity of AP and the characteristics of hospitalization (23, 24). Few studies have focused on recurrence of HTGP. Wu et al. (20) found that elevated TG levels in the ambulatory setting were associated with an increased risk of recurrent HTGP, but the study lacked TG measurements of inpatients during hospitalization for AP. The objective of this study was to analyze the clinical features of HTGP and develop a nomogram to determine the risk factors for recurrent pancreatitis in patients with HTGP.

## Methods

### Study design and participants

This was a retrospective, observational study of patients hospitalized for a first attack of HTGP admitted to the Department of Gastroenterology, First Affiliated Hospital of Nanchang University, a tertiary care referral center in China, from January 2017 to December 2019. The exclusion criteria were as follows: (a) a history of HTGP, (b) death or abandonment of treatment during the index hospitalization and incomplete hospitalization data, (c) age < 18 years, (d) chronic pancreatitis or pancreatic cancer or (e) not fulfilling 1 year follow-up. All patients were followed up with for at least 1 year after discharge, and patients who were lost to follow-up were excluded. We extracted data from a prospectively maintained database, and the use of AP data was approved by the Ethics Committee of the First Affiliated Hospital of Nanchang University (No: 2011001). Written informed consent was waived due to the retrospective nature of the study.

### Definitions

The criteria for AP diagnosis were as follows: (1) abdominal pain suggestive of AP, (2) a serum amylase and/or lipase level 3 times higher than the upper limit of normal, and (3) abdominal ultrasound, computed tomography (CT) and magnetic resonance features of AP (25). All patients met at least two of three diagnostic criteria for AP (25). HTGP was diagnosed when (1) the serum TG level was > 11.3 mmol/l or (2) the serum TG level was between 5.65 and 11.3 mmol/l without other etiologies, such as biliary or alcoholic AP (8). RAP referred to 2 or more episodes of documented AP with an interval of at least 3 months (26).

### Data collection

General baseline information, including sex, age, body mass index (BMI), history of fatty liver diseases, Charlson’s Comorbidity Index score, drinking status or smoking status, history of use for lipid-lowering medicine, time from AP attack to hospitalization and recorded data, was collected. Clinical features including the severity of pancreatitis, types of AP, hospital stay, intensive care unit (ICU) admission rate and hospitalization expense during the first hospitalization were also assessed.

Charlson’s Comorbidity Index score was used as a measure of patient comorbidity. Severity and types of pancreatitis were defined according to the revised Atlanta Classification criteria (25).

Furthermore, serum TG levels and total cholesterol (TC) levels within 24 h after an AP attack, 24 and 72 h after admission, and before discharge and high-density lipoprotein (HDL) cholesterol levels and low-density lipoprotein (LDL) cholesterol levels within 24 h after admission and before discharge were recorded. All serum tests were executed and interpreted by trained and experienced staff who were blinded to the patients’ clinical information. Serum TG, TC, HDL cholesterol, and LDL cholesterol levels were measured on an automatic biomedical analyzer (Hitachi High-Tech, 7,600–120, Japan) within 24 h. The normal reference range for TG is 0–1.7 mmol/l, TC is 0–5.7 mmol/l, HDL cholesterol is 1.29–1.55 mmol/l, and LDL cholesterol is 0–3.62 mmol/l in human serum.

Patients were followed after discharge. We collected blood lipid indexes, including serum TG, TC, HDL and LDL cholesterol levels, at 1, 6, and 12 months after discharge. The relevant follow-up time was at least 1 year; or until December 2020. A detailed clinical profile was recorded and compared between patients with or without recurrence.

### Data analysis

Categorical variables are expressed as absolute numbers and proportions, and continuous variables are described as medians and interquartile ranges (IQRs). The differences in continuous variables were analyzed by using the Kruskal–Wallis test, and the Chi-square test or Fisher’s exact test was used to compare categorical variables. Receiver operating characteristic (ROC) curve analysis was used to determine the optimal cutoff values for continuous variables. Multivariable logistic regression (a forward stepwise regression with typicality dependent variable) was further used to analyze independent risk factors for HTGP recurrence. The indicators with *p* < 0.1 in the univariate analysis were incorporated into multivariable analysis. All results are presented as odds ratios (ORs) and 95% confidence intervals (CIs). Statistical significance was considered at a *p* value of < 0.05. Statistical evaluation was performed by using SPSS software (v26.0; SPSS Inc., Chicago, IL, United States) or R software (version 4.0.5, R Development Core Team).

## Results

### Baseline characteristics and clinical profiles during index hospitalization

**Figure 1:**
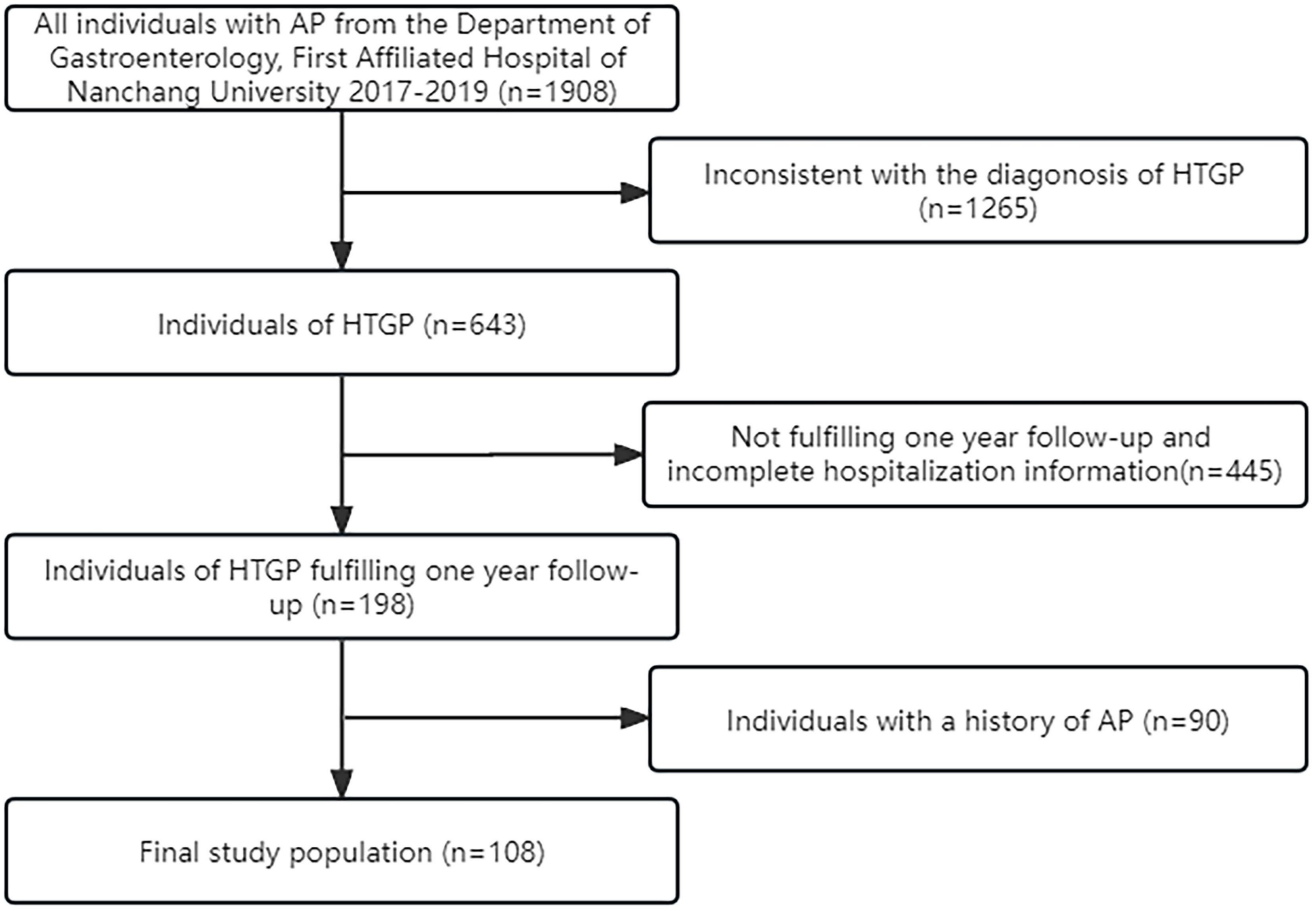
Flow-diagram for selection process of the study group of hypertriglyceridemic pancreatitis (HTGP).

A total of 108 eligible HTGP patients were included (Figure 1). Among these patients, 73.1% were male, the median age was 37 (30.344.8) years, and the mean BMI was 26.2 (24.4, 28.2) kg/m^2^. Thirty-eight patients had only one episode of AP during the observation period (nonrecurrent group), and 70 patients had at least 2 episodes of AP (recurrent group). The differences in the baseline and clinical characteristics during the index hospitalization between the two groups are summarized in Table 1. No significant difference between the two groups for baseline characteristics was observed. During the first hospitalization, milder illness was observed in patients with recurrence than in those without recurrence, as indicated by a lower proportion of necrotizing pancreatitis (11.4 vs. 31.6%; *p* = 0.010), severe acute pancreatitis (14.3 vs. 34.2%; *p* = 0.047), shorter hospital stay [8.0 (5.0, 13.5) days vs. 13 (6.5, 21.0) days; *p* = 0.040], and lower hospital costs [16,126 (8,801,36,284) RMB vs. 43,630 (10,781,111,400) RMB; *p* = 0.006].

**Table 1:** Baseline characteristics and clinical profile during index hospitalization of patients with HTGP.

	Total (*N* = 108)	Recurrent group (*n* = 70)	Non-recurrent group (*n* = 38)	*p* Value
Baseline characteristics				
Sex				0.584
Male	79 (73.1%)	50 (71.4%)	29 (76.3%)	
Female	29 (26.9%)	20 (28.6%)	9 (23.7%)	
Age, years	37.0 (30.3,44.8)	34.0 (27.8,44.3)	39.5 (32.0,45.3)	0.129
BMI, kg/m^2^	26.2 (24.4,28.2)	26.6 (24.8,28.4)	24.9 (23.6,26.8)	0.082
Charlson’s Comorbidity Index score, points	1 (0,1)	1 (1,1)	1 (0,1)	0.116
History of fatty liver disease	78 (72.2%)	51 (72.9%)	27 (71.1%)	0.842
History of diabetes	16 (14.8%)	12 (17.1%)	4 (10.5%)	0.355
History of use for lipid-lowering medicine	30 (27.8%)	19 (27.1%)	11 (28.9%)	0.842
Smoking status	42 (38.9%)	28 (40.0%)	14 (36.8%)	0.748
Drinking status	45 (41.7%)	25 (35.7%)	20 (52.6%)	0.089
Time from AP attack to admission	3.0 (2.0,4.0)	2.5 (2.0,4.0)	4.0 (2.0,5.0)	0.079
Clinical profile during index hospitalization				
Respiratory failure	43 (39.8%)	23 (32.9%)	20 (52.6%)	**0.045**
Renal failure	8 (7.4%)	3 (4.3%)	5 (13.2%)	0.126
Cardiovascular failure	7 (6.5%)	2 (2.9%)	5 (13.2%)	0.094
^*^Types of AP				**0.010**
Interstitial oedematous pancreatitis	88 (81.5%)	62 (88.6%)	26 (68.4%)	
Necrotizing pancreatitis	20 (18.5%)	8 (11.4%)	12 (31.6%)	
^*^Severity of AP				**0.047**
MAP	24 (22.2%)	18 (25.7%)	6 (15.8%)	
MSAP	61 (56.5%)	42 (60.0%)	19 (50.0%)	
SAP	23 (21.3%)	10 (14.3%)	13 (34.2%)	
Hospital stay, day	8.5 (6,17)	8.0(5.0,13.5)	13 (6.5,21.0)	**0.040**
ICU admission	44 (40.7%)	25 (35.7%)	19 (50.0%)	0.149
Hospital costs, RMB	19,953 (9,778,57,783)	16,126 (8,801,36,284)	43,630 (10,781,111,400)	**0.006**

Date are median (interquartile ranges) or absolute numbers (proportions). HTGP, hypertriglyceridemic pancreatitis; BMI, body mass index; AP, acute pancreatitis; MAP, mild acute pancreatitis; MSAP, moderately severe acute pancreatitis; SAP, severe acute pancreatitis; ICU: intensive care unit. ^*^Types and severity of AP were assessed according to 2012 Atlanta Classification of AP. *p* < 0.05 was bolded.

### Serum lipid levels and lipid-lowering treatment in patients with HTGP during the index hospitalization

**Figure 2:**
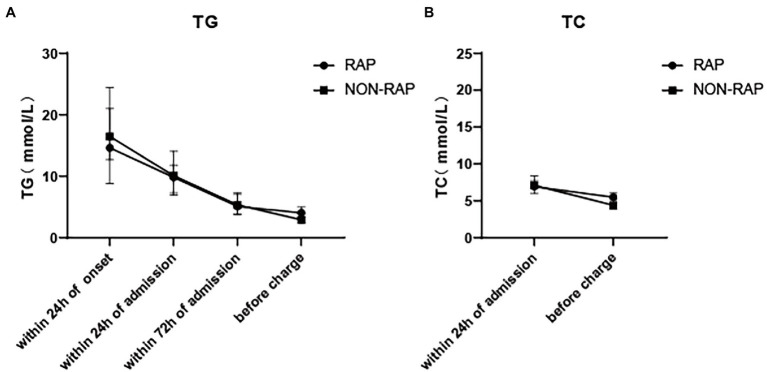
Dynamic change of serum lipid levels of patients with HTGP during index hospitalization, **(A)** the serum TG levels, **(B)** the serum TC levels.

The levels of serum TG, TC, HDL, and LDL cholesterol at different time points during hospitalization were compared between the two groups. Table 2 shows the lipid indexes and rate of lipid-lowering treatment in patients with HTGP during the first hospitalization. As the time from the onset of AP increased, the levels of serum TG and TC gradually decreased (Figure 2). The serum TG level before discharge was much lower than the serum TG level within 24 h of onset (3.7 (2.6–5.7) mmol/L vs. 16.2 (10.6–24.4) mmol/L), and the TC level followed a similar trend [9.3 (6.1–12.3) mmol/L vs. 5.1 (3.9–6.5) mmol/L], see in Figure 1. In the recurrent group, serum TG levels [4.1 (2.8–6.3) mmol/L vs. 2.9 (2.2–4.2) mmol/L, *p* = 0.002] and TC levels [5.5 (4.1–6.8) mmol/L vs. 4.4 (3.7–7.0) mmol/L, *p* = 0.031] before discharge were higher than those in the nonrecurrent group. No significant differences in the HDL and LDL cholesterol levels were found between the two groups at different time points. The majority of patients received lipid-lowering therapy. Over half of the patients were treated with oral lipid-lowering drugs, intravenous insulin, or low weight molecular heparin. Only 5.6% of the patients received hemofiltration or plasmapheresis therapy.

**Table 2:** Serum lipid levels and lipid-lowering treatments of patients with HTGP during index hospitalization.

	Recurrent group (*n* = 70)	Non-recurrent group (*n* = 38)	*p* Value
*Serum lipid levels of patients during index hospitalization*			
TG (within 24 h of onset), mmol/L	14.6 (8.7,23.6)	16.5 (12.6,24.5)	0.356
TG (within 24 h of admission), mmol/L	9.8 (5.4,14.6)	10.1 (5.9,16.4)	0.687
TG (within 72 h of admission), mmol/L	5.1 (3.1,8.2)	5.3 (3.5,7.9)	0.862
TG (before discharge), mmol/L	4.1 (2.8,6.3)	2.9 (2.2,4.2)	**0.002**
TC (within 24 h of onset), mmol/L	8.0 (5.5,11.6)	10.3 (6.9,16.2)	0.086
TC (within 24 h of admission), mmol/L	6.9 (4.8,8.3)	7.5 (5.7,11.3)	0.150
TC (within 72 h of admission), mmol/L	6.3 (4.7,7.1)	6.4 (4.3,7.9)	0.599
TC (before discharge), mmol/L	5.5 (4.1,6.9)	4.4 (3.7,6.0)	**0.031**
HDL cholesterol (within 24 h of admission), mmol/L	0.7 (0.5,1.0)	0.7 (0.5,0.9)	0.716
HDL cholesterol (before discharge), mmol/L	0.7 (0.5,0.9)	0.6 (0.5,0.8)	0.340
LDL cholesterol (within 24 h of admission), mmol/L	1.0 (0.6,3.2)	1.4 (0.6,2.8)	0.786
LDL cholesterol (before discharge), mmol/L	2.4 (1.2,3.8)	2.6 (1.3,3.2)	0.825
*Lipid-lowering treatments of patients during index hospitalization*			
Any lipid-lowering treatments	65 (92.9%)	37 (97.4%)	0.309
Oral lipid-lowering drugs	48 (68.6%)	29 (76.3%)	0.396
Intravenous insulin	56 (80.0%)	33 (86.8%)	0.372
Low molecular weight heparin	40 (57.1%)	20 (52.6%)	0.652
Hemofiltration or plasmapheresis	3 (4.3%)	3 (7.9%)	0.663

Date are median (interquartile ranges) or absolute numbers (proportions). HTGP, hypertriglyceridemic pancreatitis; TG, triglyceride; TC, total cholesterol; HDL cholesterol, high-density lipoprotein cholesterol; LDL cholesterol, low-density lipoprotein cholesterol. Normal ranges: TG (0–1.7 mmol/l), TC (0–5.7 mmol/l), HDL cholesterol (1.29–1.55 mmol/l), VDL cholesterol (0–3.62 mmol/l). *p* < 0.05 was bolded.

### Serum lipid levels of patients with HTGP during follow-up

Table 3 lists the blood lipid profiles of the two groups at different time points during the one-year follow-up. Serum TG levels and TC levels gradually increased in all two groups. Serum TG level in the recurrent group at 1 month [3.7 (2.3–9.7) vs. 2.0(1.4–2.7), *p* = 0.001], 6 months [6.1(3.1–13.1) vs. 2.5(1.1–3.5), *p* = 0.003] and 12 months [9.6(3.5–20.0) vs. (2.7(1.6–5.5), *p* = 0.001] of follow-up were higher than those in the nonrecurrent group. Similarly, the TC levels at 1 month [5.3 (4.2–6.2) vs. 4.4(4.0–4.8), *p* = 0.005], 6 months [5.4 (4.7–7.8) vs. 4.7(4.3–5.4), *p* = 0.003], and 12 months [6.6 (5.4–10.7) vs. 4.7(4.3–5.4), *p* = 0.001] of follow-up were higher than those in the nonrecurrent group.

**Table 3:** Serum lipid levels of patients with HTGP during follow-up.

After discharge	Recurrent group (*n* = 70)	Non-recurrent group (*n* = 38)	*p* Value
1 month			
TG	3.7 (2.3,9.7)	2.0 (1.4,2.7)	**0.001**
TC	5.3 (4.2,6.2)	4.4 (4.0,4.8)	**0.005**
HDL cholesterol	1.0 (0.8,1.2)	1.0 (0.9,1.2)	0.856
VDL cholesterol	2.3 (1.7,3.0)	2.7 (2.1,2.9)	0.371
6 months			
TG	6.1 (3.1,13.1)	2.5 (1.1,3.5)	**0.003**
TC	5.4 (4.7,7.8)	4.7 (4.3,5.4)	**0.019**
HDL cholesterol	0.9 (0.8,1.1)	1.1 (1.0,1.3)	0.189
LDL cholesterol	2.2 (1.5,2.8)	2.5 (2.1,2.9)	0.177
12 months			
TG	9.6 (3.5,20.0)	2.7 (1.6,5.5)	**0.001**
TC	6.6 (5.4,10.7)	4.7 (4.3,5.4)	**0.001**
HDL cholesterol	0.9 (0.7,1.1)	1.1 (1.0,1.5)	**0.009**
LDL cholesterol	1.3 (0.8,2.6)	2.5 (2.1,3.0)	**0.004**

Date are median (interquartile ranges) or absolute numbers (proportions). HTGP, hypertriglyceridemic pancreatitis; TG, triglyceride; TC, total cholesterol; HDL cholesterol, high-density lipoprotein cholesterol; LDL cholesterol, low-density lipoprotein cholesterol. Normal ranges: TG (0–1.7 mmol/l), TC (0–5.7 mmol/l), HDL cholesterol (1.29–1.55 mmol/l), LDL cholesterol (0–3.62 mmol/l). *p* < 0.05 was bolded.

**Figure 3:**
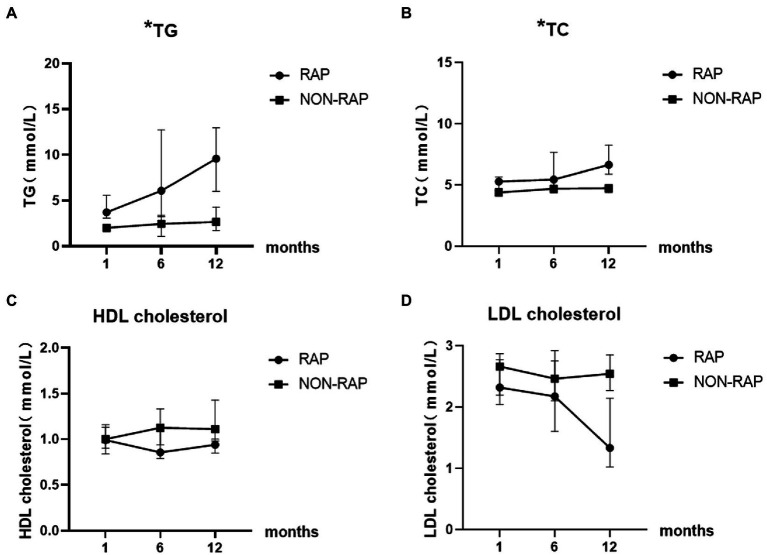
Dynamic change of serum lipid levels of patients with HTGP during follow-up, **(A)** the serum TG levels, **(B)** the serum TC levels, **(C)** the serum HDL cholesterol levels, **(D)** the serum LDL cholesterol levels. ^*^ indicates statistically significant difference.

There was no significant difference between the two groups in HDL cholesterol levels and LDL cholesterol levels at 1 month and 6 months of follow-up. At the 12-month follow-up, the levels of HDL [1.1(1.0–1.5) vs. 0.9(0.7–1.1), *p* = 0.009] and LDL [2.5(2.1–3.0) vs. 1.3(0.8–2.6), *p* = 0.004] cholesterol in the nonrecurrent group were higher than those in the recurrent group. The HDL cholesterol level was a protective factor against cardiovascular diseases, and it may also play an important role in pancreatitis, which can explain the change in the nonrecurrent group. LDL cholesterol levels were all in the normal range in both groups at different time points of follow-up, so it was not clinically meaningful. Figure 3 describes the changing trends of the levels of serum TG, TC, HDL and LDL cholesterol at different follow-up times in two groups.

### Risk factors for recurrence of HTGP

We performed ROC analysis to determine the optimal cutoff values of continuous numerical variables for predicting recurrent episodes of HTGP. Univariate (Table 4) and multivariate (Table 5) logistic regression analyses were conducted to determine the independent risk factors for recurrence of HTGP. A serum TG level > 3.1 mmol/l at 1 month of follow-up was associated with a substantially increased risk, with an adjusted rate ratio of 10.3 (95% confidence limit, 95% CI, 2.5–34.1), for recurrent episodes in patients with HTGP. Compared with individuals with a Charlson’s Comorbidity Index score less than 2 points, the multivariable adjusted OR for recurrence of HTGP was 9.0 (95% CI, 1.1–96.9) in individuals with a Charlson’s Comorbidity Index score greater than or equal to 2 points. See in Table 6.

**Table 4:** Univariable logistic regression analysis for recurrence in patients with HTGP.

	Unadjusted OR,95%CI	*p* Value
Sex (Ref: male)		
Female	1.3 (0.5–3.2)	0.585
Age (Ref: ≤ 34 years)		
> 34	0.4 (0.2–1.0)	**0.048**
BMI (Ref: < 26.8 kg/m^2^)		
≥ 26.8	3.7 (1.3–10.5)	**0.015**
Charlson’s Comorbidity Index score (Ref: < 2 points)		
≥ 2	3.5 (0.9–12.7)	0.062
History of fatty liver disease (Ref: no)	1.1 (0.5–2.6)	0.842
Yes		
History of use for lipid-lowering medicine (Ref: no)	0.9 (0.4–2.2)	0.842
Yes		
Smoking status (Ref: no)		
Yes	1.1 (0.5–2.6)	0.748
Drinking status (Ref: no)		
Yes	0.5 (0.2–1.1)	0.091
TG before discharge (Ref: TG ≤ 3.2 mmol/l)		
> 3.2	4.0 (1.7–9.3)	**0.001**
TC before discharge (Ref: TC ≤ 5.4 mmol/l)		
> 5.4	3.2 (1.3–7.5)	**0.009**
HDL cholesterol before discharge (Ref: HDL HDL ≤ 0.7 mmol/l)		
> 0.7	1.9 (0.8–4.5)	0.123
LDL cholesterol before discharge (Ref: VDL ≤ 3.3 mmol/l)		
> 3.3	2.6 (1.0–7.2)	0.063
TG at 1 month of follow-up (Ref: TG ≤ 3.1 mmol/l)		
> 3.1	11.9 (3.5–39.6)	**0.001**
TC at 1 month of follow-up (Ref: TC ≤ 5.4 mmol/l)		
> 5.4	15.2 (3.2–71.1)	**0.001**
HDL cholesterol at 1 month of follow-up (Ref: ≤ 0.9 mmol/l)		
> 0.9	0.4 (0.1–1.2)	0.091
LDL cholesterol at 1 month of follow-up (Ref: ≤ 2.1 mmol/l)		
> 2.1	0.4 (0.1–1.2)	0.110

HTGP, hypertriglyceridemic pancreatitis; OR, odds ratio; CI, confidence interval; BMI, body mass index; TG, triglyceride; TC, total cholesterol; HDL cholesterol, high-density lipoprotein cholesterol; LDL cholesterol, low-density lipoprotein cholesterol. *p* < 0.05 was bolded.

**Table 5:** Multivariate logistic regression analysis for recurrence in patients with HTGP.

	aOR,95%CI	*p* Value
Age (Ref: ≤ 34 years)		
> 34	0.7 (0.1–3.1)	0.596
BMI (Ref: < 26.8 kg/m^2^)		
≥ 26.8	2.7 (0.6–12.0)	0.188
Charlson’ Comorbidity Index score (Ref: < 2 points)		
≥ 2	9.0 (1.1—96.9)	**0.041**
Drinking status (Ref: no)		
Yes	0.8 (0.2–3.5)	0.790
TG at 1 month of follow-up (Ref: ≤ 3.1 mmol/l)		
> 3.1	10.3 (2.5–34.1)	**0.001**

Only results with *p* < 0.05 in the multivariate regression were listed above. HTGP, hypertriglyceridemic pancreatitis; aOR, adjusted odds ratio; CI, confidence interval; TG, triglyceride. *p* < 0.05 was bolded.

**Table 6:** Variables included in risk-score model and probability for recurrence of HTGP.

	TG ≤ 3.1 mmol/l	TG > 3.1 mmol/l
CCI < 2 points	0.35	0.82
CCI ≥ 2 points	0.71	0.95

“CCI” refers to Charlson’ Comorbidity Index score. “TG” refers to TG at 1 month of follow-up.

### Clinical characteristics of the recurrent group

During the follow-up period, 70 patients (64.8%) had recurrent pancreatitis, and 37% of the patients with HTGP experienced 2 or more attacks of recurrent pancreatitis. We performed a subgroup analysis in patients with recurrence, and the clinical profile was compared between index hospitalization and rehospitalization in those patients. There was a significantly higher incidence of ICU admission (35.7 vs. 17.1%, *p* = 0.009) and more severe disease (*p* = 0.044) during the index hospitalization than during rehospitalization. In addition, the length of hospitalization (8.0(5.0–13.5) vs. 6.0(4.0–9.0), *p* = 0.001) was longer and the hospital costs [16,126 (8801–36,284) vs. 11,319 (6,243,23,111), *p* = 0.029] were higher during the index hospitalization than during rehospitalization. Comparisons of blood lipid indexes, treatments and clinical outcomes in the recurrent group in terms of first admission and readmission are detailed in Table 7.

**Table 7:** Clinical profile compared between index and recurrent hospitalization in patients with recurrence.

	Index hospitalization (*n* = 70)	Recurrent hospitalization (*n* = 70)	*p* Value
*Serum lipid levels*			
TG (within 24 h of onset), mmol/L	14.6 (8.7,23.6)	26.1 (12.1,36.4)	0.510
TG (within 24 h of admission), mmol/L	9.8 (5.4,14.6)	9.3 (4.5,22.1)	0.227
TG (within 72 h of admission), mmol/L	5.1 (3.1,8.2)	6.0 (3.7,9.8)	0.221
TG (before discharge), mmol/L	4.1 (2.8,6.3)	4.9 (3.2,6.7)	0.232
TC (within 24 h of onset), mmol/L	8.0 (5.5,11.6)	11.4 (6.8,15.8)	0.878
TC (within 24 h of admission), mmol/L	6.9 (4.8,8.3)	6.9 (4.9,11.0)	0.085
TC (within 72 h of admission), mmol/L	6.5 (4.6,7.8)	6.3 (4.7,7.1)	0.782
TC (before discharge), mmol/L	5.5 (4.1,6.9)	5.4 (4.5,7.1)	0.840
HDL cholesterol (within 24 h of admission), mmol/L	0.7 (0.5,1.0)	0.9 (0.6,1.2)	0.017
HDL cholesterol (before discharge), mmol/L	0.7 (0.5,0.9)	0.7 (0.6,0.9)	0.315
LDL cholesterol (within 24 h of admission), mmol/L	1.0 (0.6,3.2)	1.2 (0.6,2.3)	0.383
LDL cholesterol (before discharge), mmol/L	2.4 (1.2,3.8)	2.6 (1.7,3.5)	0.777
*Clinical treatment and outcome*			
Time from AP attack to admission	2.5 (2.0,4.0)	2.0 (2.0,3.0)	0.209
Any lipid-lowering treatment	65 (92.9%)	63 (90.0%)	
Oral lipid-lowering drugs	48 (68.6%)	43 (61.4%)	0.369
Intravenous insulin	56 (80.0%)	51 (72.9%)	0.297
Low molecular weight heparin	40 (57.1%)	39 (55.7%)	0.869
Hemofiltration or plasmapheresis	3 (4.3%)	1 (1.4%)	0.317
^*^Severity of AP			**0.044**
MAP	18 (25.7%)	27 (38.6%)	
MSAP	42 (60.0%)	38 (54.3%)	
SAP	10 (14.3%)	5 (7.1%)	
Hospital day, day	8.0 (5.0,13.5)	6.0 (4.0,9.0)	**0.001**
ICU admission	25 (35.7%)	12 (17.1%)	**0.009**
Hospital costs, RMB	16,126 (8,801,36,284)	11,319 (6,243,23,111)	**0.029**

Date are median (interquartile ranges) or absolute numbers (proportions). HTGP, hypertriglyceridemic pancreatitis; TG, triglyceride; TC, total cholesterol; HDL cholesterol, high-density lipoprotein cholesterol; LDL cholesterol, low-density lipoprotein cholesterol. Normal ranges: TG (0–1.7 mmol/l), TC (0–5.7 mmol/l), HDL cholesterol (1.29–1.55 mmol/l), LDL cholesterol (0–3.62 mmol/l). AP, acute pancreatitis; MAP, mild acute pancreatitis; MSAP, moderately severe acute pancreatitis; SAP, severe acute pancreatitis. ^*^Severity of AP were assessed according to 2012 Atlanta Classification of AP. ICU: intensive care unit. *p* < 0.05 was bolded.

## Discussion

We retrospectively studied the baseline clinical features and lipid levels of patients with HTGP and assessed the dynamic change in serum TG levels within 1 year after the first attack of HTGP. Analysis of these data showed that a high Charlson’s Comorbidity Index score was related to recurrent episodes of HTGP. Charlson’s Comorbidity Index score is an extensively used comorbidity index. To date, few studies have reported that the Charlson’s Comorbidity Index score is a risk factor for recurrence of HTGP. Of our study’s findings, a high Charlson’s Comorbidity Index score was associated with an increased risk of recurrence in patients with HTGP, which may suggest that we should focus on patients with more comorbidities.

One finding of our study on the risk factors for recurrent HTGP is consistent with those of previous studies. The serum TG level at 1 month of follow-up was closely associated with HTGP recurrence. Some reports have suggested that serum TG levels after hospital discharge higher than 5.65 mmol/l are independently associated with recurrent pancreatitis (27, 28). Even moderately elevated baseline serum TG levels (2.25 mmol/l-5.65 mmol/l) within 12 months postdischarge were associated with a significant increase in the risk of disease recurrence (20). In this study, we found that a serum TG level > 3.1 mmol/l at 1 month of follow-up was strongly related to the recurrence of HTGP, with an adjusted OR of 10.3, consistent with previous studies. In addition, a high Charlson’s Comorbidity Index score (≥ 2 points) was also related to an increased risk of recurrent attacks, with an adjusted OR of 9.0. On the basis of previous studies, it is usually considered that serum TG levels should be controlled below 5.65 mmol/l to prevent the recurrence of HTGP (29). However, mild elevation of serum TG levels also increase the risk of HTGP (30). The compliance of patients may also play a major role in the control of serum TG levels. More consensus is needed on serum TG level required for control.

Hypertriglyceridemia (HTG) is a well-established cause of AP (31). Zheng et al. (12) reported that the incidence of HTGP has surpassed that of alcoholic pancreatitis as the second leading cause of AP. In recent years, the incidence of HTGP has shown an increasing trend (11). Other studies have revealed that the recurrence rate of HTGP was also high and could reach up to 30.1–32% (17, 18). In this retrospective study, the recurrence rate of HTGP was 64.8%, which was much higher than the recurrence rate in previous related studies. The possible reasons may be that the follow-up period was 1 year, which caused considerable enrollment bias because some patients did not attend our hospital for regular follow-up. The recurrence rate may be overestimated.

Increased production and/or decreased catabolism of serum TG can lead to HTG. Primary HTG is usually caused by genetic abnormalities, while secondary HTG is often caused by environmental and external factors, such as diabetes, obesity, and the use of certain drugs (19, 29). The mechanism by which HTG causes HTGP to occur is not yet clear. It may be related to fatty acid toxicity, microcirculation disturbance, oxidative stress, Ca^2+^ overload and gene polymorphisms (32–39). Qi et al. (40) confirmed that the interaction between genes and the environment is extremely significant in the occurrence and development of HTGP. It is vitally important to actively control serum TG levels to reduce the occurrence of HTG and even the occurrence and recurrence of HTGP.

In the recurrent group, there was no significant difference in the blood lipid profile or lipid-lowering treatment during the two hospitalizations. As previous studies have shown no superiority in any lipid-lowering treatments of severe hypertriglyceridemia among patients hospitalized for pancreatitis (41). From the data analysis, it can be seen that the proportions of MSAP and SAP in patients in the first hospitalization were higher than those at readmission. More serious disease may lead to higher compliance of patients, thus reducing the rate of rehospitalization. However, this finding does not suggest that the severity of index hospitalization of patients with recurrence is more severe than that of rehospitalization. We did not include patients who died, which accounts for a large proportion of the severity of HTGP. In addition, according to clinical experience, the severity of HTGP in patients with relapse is increased when they are readmitted to the hospital. Thus far, there are insufficient data to support this idea. More studies are needed to explore the severity of HTGP in patients with relapse.

Our article is a retrospective study with several limitations. One is that we retrospectively collected data subject to selection bias and overrepresentation of patients to some extent. Second, these data lacked information about the time of lipid-lowering treatment, the timing of lipid-lowering drug discontinuation and other medication use during the follow-up period, which may affect the research results. In addition, as mentioned above, we did not calculate mortality, which may lead to underestimation of the severity of the disease during hospitalization and an inability to assess the impact of severity on recurrence.

## Conclusion

The present study focused on the risk factors for recurrence of HTGP. It was found that poorly controlled serum TG level at 1 month of follow-up and a high Charlson’s Comorbidity Index score (≥ 2 points) were associated with higher recurrence rates. The results of the study indicate that it is extremely important to prevent and treat underlying diseases and to control serum TG levels in patients with HTGP during follow-up.

## Data availability statement

The raw data supporting the conclusions of this article will be made available by the authors, without undue reservation.

## Ethics statement

The studies involving human participants were reviewed and approved by The institutional review boards of the First Affiliated Hospital of Nanchang University (No: 2011001). The patients/participants provided their written informed consent to participate in this study.

## Author contributions

LG collected the data, analyzed formal information, and wrote the manuscript. LD designed this study and revised the draft. JW, LX, WH, HX, LL, and NL provided guidance and amendments. YZ revised the draft and approved the final submission. All authors contributed to the article and approved the submitted version.

## Funding

This work was supported by the Double-Thousand Plan of Jiangxi Province (No. jxsq2019201028).

## Conflict of interest

The authors declare that the research was conducted in the absence of any commercial or financial relationships that could be construed as a potential conflict of interest.

## Publisher’s note

All claims expressed in this article are solely those of the authors and do not necessarily represent those of their affiliated organizations, or those of the publisher, the editors and the reviewers. Any product that may be evaluated in this article, or claim that may be made by its manufacturer, is not guaranteed or endorsed by the publisher.
